# Visual Outcomes Following Non-Diffractive Extended-Depth-of-Focus Intraocular Lens Implantation in Patients with Epiretinal Membrane in One Eye and Bilateral Cataracts †

**DOI:** 10.3390/biomedicines12112443

**Published:** 2024-10-24

**Authors:** Laura Sararols, Mercè Guarro, Meritxell Vázquez, Sergi Ruiz, Elena López, Marc Biarnés

**Affiliations:** 1 OMIQ Research, c/Tamarit 39, 08205 Sabadell, Barcelona, Spain; 2 Hospital General de Granollers, c/Frances Ribas s/n, 08402 Granollers, Barcelona, Spain

**Keywords:** cataract, epiretinal membrane, extended depth of focus, intraocular lens, pars plana vitrectomy, phacoemulsification, phacovitrectomy

## Abstract

Background/Objectives: This study aimed to characterize the visual performance in patients with bilateral cataracts and a unilateral epiretinal membrane (ERM) undergoing cataract surgery with non-diffractive extended depth of focus (EDoF) intraocular lens (IOL) placement in both eyes and phacovitrectomy in the eye with ERM. Methods: This was a prospective, descriptive, single-arm study. Visual outcomes were measured in monocular and binocular conditions in patients with bilateral cataracts and unilateral ERM stages 2 or 3 implanted with an EDoF IOL. At 6 months, visual acuity (uncorrected and corrected at different distances and contrast levels), contrast sensitivity, and visual disturbances assessed using a Light Distortion Analyzer were determined. Results: We included 22 patients (50% females, mean age of 71.4 ± 5.8 years). Mean monocular best-corrected visual acuities at 100% contrast were 0.07 ± 0.09, 0.23 ± 0.10, and 0.48 ± 0.14 logMAR for eyes with ERM, and 0.02 ± 0.08, 0.19 ± 0.11, and 0.41 ± 0.18 logMAR for fellow eyes for far, intermediate, and near distance vision, respectively. No differences were observed in visual outcomes, contrast sensitivity, or visual disturbances between ERM and non-ERM eyes, except for near distance. No unexpected adverse events were observed. Conclusions: Non-diffractive EDoF IOL can be considered in patients with cataracts and stage 2 or 3 ERM pursuing spectacle independence.

## 1. Introduction

Idiopathic epiretinal membrane (ERM) is a common macular disorder in older adults, with a prevalence of 2% to 20% [1]. Epiretinal membranes are thought to be the consequence of fibroglial proliferation on the inner surface of the retina associated with small tears in the inner limiting membrane during a posterior vitreous detachment. Anteroposterior forces result in vertical traction and increased retinal thickness. In contrast, tangential forces pull the superficial retinal layers away from their original position [2], straighten or twist retinal vessels, and reduce the foveal avascular zone [3] and macular edema [4]. As a result, patients experience metamorphopsia, micropsia, monocular diplopia, or visual acuity loss [2]. Its management includes observation or, in the 30% of cases in which there is progression [5], vitrectomy surgery, with a reasonable success rate.

Today, all patients have high expectations of enhanced vision and reduced dependence on glasses following cataract surgery. However, numerous studies have shown that multifocal intraocular lenses (IOLs) can lead to a decline in contrast sensitivity as a consequence of incident light dispersion [6,7,8,9]. Several studies [10,11], including a meta-analysis [9], have found that individual patient satisfaction is related to many factors, including the level of uncorrected visual acuity, contrast sensitivity, or halos/glare.

Recent advances in IOL designs have led to the development of an extended depth of focus (EDoF). These IOLs extend the range of focus, with the goal of offering excellent vision not only at far but also at intermediate distances, thereby also enhancing near vision. Several studies that have compared the optical quality of EDoF IOL vs. bifocal or trifocal multifocal lenses have confirmed the optimal vision quality obtained with EDoF lenses [12,13,14,15]. This illustrates that the indications for EDoF IOLs could perhaps be broadened to encompass selected cases in which contrast sensitivity is a concern, such as those with ERM, mild to moderate glaucoma, or subjects post-LASIK.

The AcrySof^®^ IQ Vivity^®^ lens (Alcon Healthcare, Fort Worth, TX, USA) is a multifocal EDoF lens that does not split the light entering the eye. Consequently, it does not compromise contrast sensitivity or cause as many dysphotopic phenomena as other multifocal IOLs [14,16]. Also, it offers excellent vision at all distances, though glasses are needed for near tasks [16].

This study assessed the visual performance after the implant of the EDoF AcrySof IQ Vivity^®^ lens in both eyes of patients with ERM in one eye and cataracts in both eyes.

## 2. Materials and Methods

This was a prospective, single-arm descriptive study. Participants were patients with surgical ERM (stages 2 or 3) in one eye and cataracts in both eyes. The eye with ERM and cataracts was first treated in two separate operations, and less than one month later, surgery was performed in the eye with cataract only. The lens implanted in both eyes was the AcrySof IQ Vivity^®^. Surgeries were carried out at the OMIQ (Oftalmologia Mèdica i Quirúrgica) Research facilities in Barcelona (Spain). The study protocol adhered to the principles outlined in the Declaration of Helsinki and received approval from the Ethics Committee of the Grupo Hospitalario Quirónsalud-Catalunya. All participants signed an informed consent form before undergoing any procedure or test.

### 2.1. Patients

Participants were required to have both bilateral cataracts and primary or idiopathic unilateral stage 2 or 3 ERM. The latter was defined as follows. Stage 2: presence of ERM associated with progressive retinal distortion, foveal depression loss, and characteristic stretching of the outer nuclear layer, with all retinal layers defined and clearly identified using optical coherence tomography (OCT). Stage 3: the presence of an ERM with continuous ectopic inner foveal layers anomalously crossing the central foveal area, foveal depression absent, and all retinal layers clearly identified using OCT [17]. Further inclusion criteria were good expected postoperative distance-corrected visual acuity (VA ≤ 0.2 logMAR or Snellen equivalent ≥ 20/32), age over 50 years, and follow-up available at 6 months post-surgery. Exclusion criteria were other concomitant posterior segment disorders; myopia < −5.00 D; presence or history of any systemic disorder, condition, or disease that could interfere with procedures or interpretation of results; previous intraocular surgery (vitrectomy in the fellow eye, glaucoma surgery, etc.); and any systemic co-morbidity which, based on the investigator’s expert medical opinion, could confound the results of the study or increase the risk for the subject.

The number of participants considered sufficient to describe the visual results expected from this study was 20. We therefore recruited 22 individuals in case some patients withdrew from the study before completing 6 months of follow-up.

### 2.2. Intraocular Lens

The AcrySof IQ Vivity^®^ lens is manufactured as a single piece utilizing a hydrophobic acrylic material with a high refractive index (*n* = 1.55). The lens is a non-diffractive EDoF IOL with wavefront-shaping X-WAVE technology that incorporates two smoothly transitioning elements designed to manipulate and redirect the wavefront. One of these elements is a slightly elevated smooth plateau (~1 µm high) that extends the wavefront, creating a continuous focal range. The other element is a curvature change (across the 2.2 mm region), effectively shifting the wavefront to maximize the utilization of all light energy. This innovative design ensures the efficient use of virtually all transmitted light energy passing through the IOL [18].

The lens anterior surface is intentionally configured with a negative spherical aberration of −0.20 µm for a 6 mm pupil, compensating for the positive spherical aberration typically found in the natural human cornea [19]. Lastly, a blue light-filtering chromophore was added to filter light similarly to the human crystalline lens and reduce the transmission of potentially harmful ultraviolet and blue light wavelengths.

### 2.3. Clinical Tests

Each participant underwent a thorough preoperative eye examination, including both objective and subjective refraction, uncorrected distance (4 m) visual acuity (UCDVA), and corrected distance VA (CDVA) using optotypes from the Early Treatment Diabetic Retinopathy Study (ETDRS test), as well as examinations of the anterior and posterior segments. Additional preoperative assessments included corneal topography (Pentacam^®^, Oculus Inc), spectral domain optical coherence tomography (OCT) of the macula and peripapillary region (Spectralis HRA+OCT^®^, Heidelberg Engineering; Heidelberg, Germany), and ocular biometry (Lenstar LS 900^®^, Haag-Streit; Köniz, Switzerland).

Intraocular lens powers were determined using the Barrett Universal II formula targeting post-surgery emmetropia in all eyes. Follow-up visits were scheduled for 1 day, 1 week, 1 month, and 3 and 6 months post-surgery. All surgeries were conducted by two experienced surgeons (LS and MG). The outcome variables for this study were measured at the 6-month visit.

Tests were carried out monocularly and binocularly. Photopic (~85 cd/m^2^) VA evaluations employed the ETDRS test at distances of 4 m, 66 cm, and 40 cm for far, intermediate, and near vision, respectively. Contrast sensitivity was gauged with the Pelli Robson test, and VA tests were conducted at various contrast levels (100% for far distance and 25% for intermediate and near distances). The Light Distortion Analyzer (LDA; Bynarytarget Lda, Braga, Portugal) was employed for non-invasive objective measurement of dysphotopsias. This device had a central white LED surrounded by 240 small white LEDs arranged in 24 hemimeridians with a separation angle of 15°, covering a 10° area at a testing distance of 2 m. Peripheral stimuli were presented to the subject from the central zone in all hemimeridians. The speed of stimulus presentation remained constant throughout.

Sitting 2 m from the device in a dark room, subjects were instructed to call out as soon as a small LED appeared differently from the central LED light. This test was repeated three times under both monocular and binocular conditions, and the result was taken as the mean of the three measures. The light distortion index (LDI) was calculated as the ratio between the area of points missed by the subject and the entire area examined, expressed as a percentage (%) of the area covered by the central LED-induced halo. The best-fit circle radius (BFCRad) was defined as the circle that best fit into the area of distortion, delineated by a line connecting all the points in each meridian. This variable is expressed in millimeters.

### 2.4. Statistical Analysis

Data analysis was performed using SPSS software for Windows version 27.0 (IBM, Armonk, NY, USA). Descriptive statistics were used to analyze the data. We used the Shapiro–Wilk test to assess the normality of each study metric. A descriptive analysis was performed, including mean and standard deviation (SD) for each variable recorded. When parametric analysis was possible, we used Student’s *t*-test for paired data to compare results between eyes with and without ERM, between consecutive visits, or between pre- and postoperative values when appropriate. When parametric analysis was not possible, we used the Wilcoxon test to compare the variables. Significance was set at *p* < 0.05 and is represented by the symbol * in the results.

## 3. Results

A total of 22 patients were enrolled, with an equal distribution between genders (50% female, *n* = 11). The mean age was 71.4 ± 5.8 years and ranged from 62 to 80 years. Half of the patients had an ERM in the right eye, and the remaining half had an ERM in the left eye. Among the ERM, 27% were classified as stage 2 (*n* = 12) and 73% as stage 3 (*n* = 32). Additionally, 55% of the ERMs (*n* = 24) were characterized as regular, and 86% (*n* = 38) showed no outer retinal changes. Following surgical ERM correction, 94% (*n* = 41) showed improvement in the ectopic inner foveal layer. Foveal recovery included 83% (*n* = 37) of individuals with stage 2 ERM with a restored foveal depression; only 31% (*n* = 14) of those with stage 3 ERM showed similar recovery at 6 months. Table 1 compares the biometric data between eyes with and without an epiretinal membrane.

Table 2 and Table 3 provide the results obtained for monocular and binocular visual acuity, respectively. At 6 months of follow up, significant differences between groups (ERM vs. non-ERM) were observed only in near vision, with those undergoing phacovitrectomy showing worse acuity (*p* ≤ 0.048). At 6 months post-surgery, only one patient had a spherical equivalent greater than 0.5 D in the non-ERM group, and two patients in the ERM group had a spherical equivalent greater than 0.5 D.

**Figure 1:**
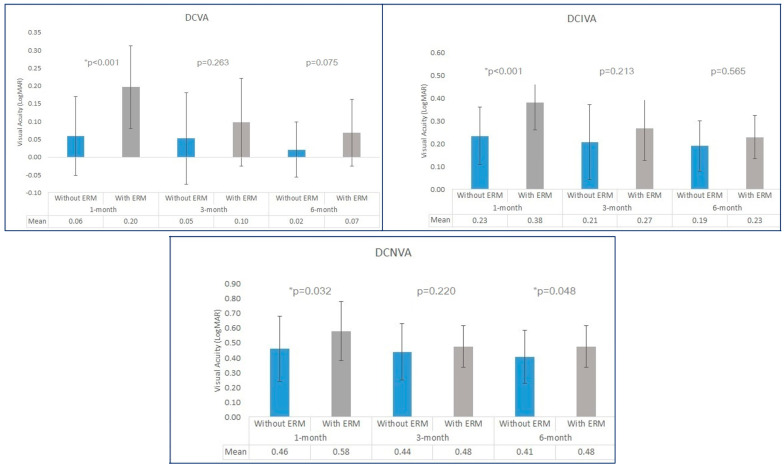
Evolution of distance, intermediate, and near visual acuities via surgical procedure at months 1, 3, and 6 post-surgery. DCIVA: distance-corrected intermediate visual acuity; DCNVA: distance-corrected near visual acuity; DCVA: distance-corrected visual acuity. *: statistically significant results (*p* < 0.05).

The evolution of visual acuity via a surgical procedure (phacoemulsification or phacovitrectomy) is shown in Figure 1. It confirms a progressive improvement in visual acuity in both groups with time but is more marked in eyes with ERM. At month 1, the visual acuities were better in eyes without ERM irrespective of the distance being evaluated, but they became similar at 3 and 6 months and were only different in the near range.

When comparing contrast sensitivity between eyes with and without ERM, no significant differences were found (Table 4). Similarly, no differences were observed in visual disturbances between the two groups of eyes, as indicated in Table S1.

## 4. Discussion

The implant of a multifocal intraocular lens in adults with a retinal disorder may be considered a contraindication owing to a possible decrease in contrast sensitivity post-surgery despite a good visual acuity outcome [20]. However, it should be noted that risk factors for retinal and cataract disease considerably overlap, indicating that these two conditions often co-occur in older adults [21].

Studies examining the behavior of diffractive multifocal lenses have shown that in persons with ERM, these lenses result in poor refractive outcomes, involving reductions in visual acuity and contrast sensitivity [22,23]. Fortunately, the introduction of new generation lenses, such as the AcrySof IQ Vivity^®^ lens, which does not split light, has improved outcomes in these patients, including contrast sensitivity comparable to that achieved with monofocal lenses [24]. This indicates that for persons with ocular diseases like glaucoma or a retinal disorder, the placement of this type of lens will enable them to have spectacle-free vision at various distances with no increase in dysphotopic phenomena.

In a recent study, Jeon et al. [25] analyzed the performance of the AcrySof IQ Vivity^®^ lens in patients with epiretinal membranes. However, their study was retrospective, and ERMs were limited to stages 1 and 2. In contrast, our prospective study explores more advanced stages (2 and 3) and examines the bilateral implant of these lenses. With this approach, we were able to effectively compare outcomes in eyes with and without ERMs.

Six months after the IOL implant, no significant or clinically relevant differences in visual acuity for distance and intermediate vision (66 cm) were noted between eyes with and without ERMs. However, for near vision, better results were obtained in eyes without ERMs, both with and without distance correction. Although significant, the difference was less than one line of vision. The results of Jeon et al. [25], also obtained six months after IOL implant, indicated better intermediate vision (VA 0.14 vs. 0.22) and near vision (VA 0.23 vs. 0.46). However, these results were only presented without correction, and postoperative refractive defects were not specified. Accordingly, a slight myopic defect could explain this difference. Chung et al. [26] examined an advanced monofocal lens, the Tecnis ICB00 model (Johnson & Johnson Vision Care Inc., Irvine, CA, USA), in patients with ERMs. Patients were divided into two groups: Group 1 included stage 1 and 2 ERM, while Group 2 included stage 3. Outcomes in the two groups in terms of distance vision were similar to those obtained in our study. However, our outcomes were better in that there were differences in intermediate vision (0.26 for Group 1 and 0.32 for Group 2, compared to 0.22) and more pronounced differences in near vision (0.71 for Group 1 and 0.78 for Group 2, compared to 0.46).

When comparing our binocular visual results with those obtained in other studies performed on healthy eyes, we find consistent VA to that reported by Gundersen et al. [27] for far (−0.07 vs. −0.01) and intermediate distance (0.00 vs. 0.10). However, our near distance acuities were less favorable (0.07 vs. 0.28), though comparable, to those obtained by Bala et al. [28] (0.31). In any case, our monocular results also indicated differences between the two groups of eyes in near vision. However, there was an improvement due to binocular summation compared to the results obtained monocularly in the eye without an ERM (0.28 vs. 0.37).

As mentioned earlier, adequate contrast sensitivity is crucial in patients with retinal disease, as this factor may be impacted by the condition. Our study revealed no contrast sensitivity difference between the eye with an ERM and the fellow eye in measurements conducted with the Pelli-Robson test or with a low-contrast test (25%) at various distances. Jeon et al. [25] observed worse contrast sensitivity in eyes with ERMs, speculating that the impaired contrast sensitivity in their ERM group might be attributed to the membrane itself and the disorganization of underlying retinal cells, disrupting the penetration of light. In our study, the agreement observed between eyes with and without ERMs, despite a more advanced stage of membrane development, could be attributed to the good postoperative outcomes observed at the retinal level, whereby 94% of cases showed improvement in the ectopic inner foveal layer.

We also compared visual disturbances between eyes with and without ERMs. Hence, although the eyes without a membrane showed slightly better results, differences between the two groups were not significant or clinically relevant. The literature lacks data on this variable with which to compare our results.

The limitations of our study include the size of the analyzed sample. Given that this was a pilot study, the sample size was not determined. Further research is needed to calculate the sample size needed to detect specific differences between the study groups based on these results. Another limitation is that there was no control group of individuals with both eyes healthy, aside from cataracts, allowing for the direct comparison of data, particularly with regard to the binocular data collected.

Our results indicate that eyes affected by stage 2 or 3 fovea-involving ERMs could benefit from combined phacovitrectomy with the implantation of non-diffractive EDoF IOL with X-Wave technology. No differences were observed in visual outcomes, contrast sensitivity, or the presence of visual disturbances, except for near visual acuity when compared to eyes without ERM. Based on our findings, the AcrySof IQ Vivity^®^ lens appears to be a viable option for patients with unilateral ERM seeking spectacle independence, irrespective of foveal involvement. The limitations of our pilot study underscore the need for larger-scale research to validate these findings and optimize clinical applications.

## Figures and Tables

**Table 1:** Monocular descriptive preoperative data. CMT: central macular thickness; D: diopters; Km: mean keratometry; ERM: epiretinal membrane; FCP: foveal center point; IOP: intraocular pressure; µm: microns; mm: millimeters; mmHg: millimeters of mercury; SD: standard deviation; SE: spherical equivalent; RNFL: retinal nerve fiber layer. * Statistically significant difference.

	Eyes with ERM Mean ± SD (Range)	Eyes Without ERM Mean ± SD (Range)	*p*-Value
Preoperative SE (D)	0.90 ± 1.56 (−3.75 to 3.50)	0.85 ± 1.36 (−1.75 to 3.50)	0.995
Preoperative Km (D)	43.67 ± 1.50 (40.84 to 45.78)	43.95 ± 1.62 (40.94 to 46.84)	0.208
Axial length (mm)	23.43 ± 0.69 (22.30 to 25.17)	23.40 ± 0.71 (22.02 to 24.81)	0.907
Anterior chamber depth (mm)	3.05 ± 0.28 (2.66 to 3.69)	3.09 ± 0.29 (2.60 to 3.64)	0.803
Implanted IOL (D)	21.77 ± 1.93 (18.50 to 27.00)	21.09 ± 1.01 (20.00 to 23.50)	0.07
Preoperative IOP (mmHg)	14.18 ± 2.92 (10.00 to 20.00)	14.27 ± 2.60 (10.00 to 21.00)	0.507
Pre-CMT (µm)	423.32 ± 80.87 (289.00 to 564.00)	282.45 ± 30.59 (185.00 to 337.00)	<0.001 *
Pre-RNFL (µm)	103.74 ± 12.46 (81.00 to 126.00)	99.16 ± 9.06 (79.00 to 111.00)	0.353
Pre-FCP (µm)	399.91 ± 101.41 (211.00 to 567.00)	236.36 ± 36.44 (146.00 to 337.00)	<0.001 *
Pre-volume (mm^3^)	9.85 ± 1.25 (8.42 to 12.81)	8.57 ± 0.46 (7.73 to 9.42)	<0.001 *

**Table 2:** Monocular visual outcomes at 6 months post-surgery in eyes with and without ERM in logMAR scale. CDVA: corrected distance visual acuity; DCIVA: distance-corrected intermediate visual acuity; DCNVA: distance-corrected near visual acuity; ERM: epiretinal membrane; SD: standard deviation; UDVA: uncorrected distance visual acuity; UIVA: uncorrected intermediate visual acuity; UNVA: uncorrected near visual acuity. * Significant difference.

	Eyes with ERM Mean ± SD (Range)	Eyes Without ERM Mean ± SD (Range)	*p*-Value
Preoperative monocular CDVA	0.24 ± 0.18 (0.00 to 0.74)	0.10 ± 0.10 (0.00 to 0.38)	0.002 *
Postoperative monocular UDVA, 6 months	0.12 ± 0.12 (−0.02 to 0.36)	0.09 ± 0.14 (−0.12 to 0.42)	0.288
Postoperative monocular CDVA, 6 months	0.07 ± 0.09 (−0.02 to 0.36)	0.02 ± 0.08 (−0.12 to 0.20)	0.075
Postoperative monocular UIVA, 6 months	0.22 ± 0.09 (0.02 to 0.34)	0.18 ± 0.10 (0.02 to 0.36)	0.991
Postoperative monocular DCIVA, 6 months	0.23 ± 0.10 (0.08 to 0.40)	0.19 ± 0.11 (0.02 to 0.40)	0.565
Postoperative monocular UNVA, 6 months	0.46 ± 0.13 (0.14 to 0.78)	0.37 ± 0.18 (0.10 to 0.70)	0.037 *
Postoperative monocular DCNVA, 6 months	0.48 ± 0.14 (0.16 to 0.78)	0.41 ± 0.18 (0.10 to 0.70)	0.048 *

**Table 3:** Binocular visual acuity values at 6 months post-surgery in logMAR scale. CDVA: corrected distance visual acuity; DCIVA: distance-corrected intermediate visual acuity; DCNVA: distance-corrected near visual acuity; SD: standard deviation; UDVA: uncorrected distance visual acuity; UIVA: uncorrected intermediate visual acuity; UNVA: uncorrected near visual acuity.

	Binocular Values Mean ± SD (Range)
Preoperative binocular CDVA	0.24 ± 0.18 (0.00 to 0.74)
Postoperative binocular UDVA, 6 months	0.03 ± 0.13 (−0.16 to 0.52)
Postoperative binocular CDVA, 6 months	−0.01 ± 0.07 (−0.16 to 0.20)
Postoperative binocular UIVA, 6 months	0.10 ± 0.08 (−0.08 to 0.24)
Postoperative binocular DCIVA, 6 months	0.10 ± 0.08 (−0.02 to 0.24)
Postoperative binocular UNVA, 6 months	0.28 ± 0.15 (0.04 to 0.60)
Postoperative binocular DCNVA, 6 months	0.30 ± 0.15 (0.04 to 0.60)

**Table 4:** Monocular contrast sensitivity measured at 6 months post-surgery in eyes with and without ERM. Cm: centimeters; SD: standard deviation; VA: visual acuity.

	Eyes with ERM Mean ± SD (Range)	Eyes Without ERM Mean ± SD (Range)	*p*-Value
Postoperative monocular Pelli-Robson	1.51 ± 0.12 (1.35 to 1.65)	1.55 ± 0.11 (1.35 to 1.65)	0.834
Postoperative monocular VA, low contrast (25%), 66 cm	0.71 ± 0.13 (0.44 to 1.00)	0.67 ± 0.18 (0.36 to 1.00)	0.089
Postoperative monocular VA, low contrast (25%), 40 cm	0.80 ± 0.17 (0.54 to 1.10)	0.73 ± 0.18 (0.44 to 1.04)	0.970

## Data Availability

Data may be available upon reasonable request and after approval by the sponsor.

## Supplementary Materials

The following supporting information can be downloaded at: https://www.mdpi.com/article/10.3390/biomedicines12112443/s1, Table S1: Monocular visual disturbance measurements obtained in psychophysical tests at 6 months post-surgery in eyes with and without ERM.
