# Supplementary material for: Visual Outcomes Following Non-Diffractive Extended-Depth-of-Focus Intraocular Lens Implantation in Patients with Epiretinal Membrane in One Eye and Bilateral Cataracts
Source: Biomedicines. 2024 Oct 24;12(11):2443. doi: 10.3390/biomedicines12112443 (PMC11591685; doi:10.3390/biomedicines12112443)
Supplement: Supplementary file 1 [file biomedicines-12-02443-s001.zip › biomedicines-3196098-supplementary.pdf]

Table S1. Monocular visual disturbance measurements obtained in psychophysical tests at 6 months post-surgery in eyes with and without ERM. BFC irregular: best-fit circle radius irregularity; BFCRad: best-fit circle radius; ERM: epiretinal membrane; LDI: light distortion index; mm: millimetres; SD: standard deviation.

	**Eyes with ERM Mean ± SD (range)**	**Eyes without ERM Mean ± SD (range)**	***p*-Value**
Postoperative monocular LDI (%) 6 months	14.38 ± 13.62 (3.82 to 68.44)	13.81 ± 15.88 (4.77 to 81.17)	0.833
Postoperative monocular BFCRad (%) 6 months	29.20 ± 10.91 (16.00 to 68.17)	27.87 ± 11.92 (18.00 to 74.00)	0.752
Postoperative monocular BFC irregular (mm) 6 months	0.71 ± 1.04 (0.00 to 4.83)	0.49 ± 0.34 (0.00 to 1.24)	0.054
